# Macrophage Inhibitory Cytokine-1 (*MIC-1/GDF15*) Gene Deletion Promotes Cancer Growth in TRAMP Prostate Cancer Prone Mice

**DOI:** 10.1371/journal.pone.0115189

**Published:** 2015-02-19

**Authors:** Yasmin Husaini, Glen P. Lockwood, Trung V. Nguyen, Vicky Wang-Wei Tsai, Mohammad G. Mohammad, Pamela J. Russell, David A. Brown, Samuel N. Breit

**Affiliations:** 1 St. Vincent’s Centre for Applied Medical Research, St. Vincent’s Hospital and University of New South Wales, Sydney, NSW 2010, Australia; 2 Australian Prostate Cancer Research Centre-Queensland, Institute of Health and Biomedical Innovation, Queensland University of Technology, Translational Research Institute, Brisbane, QLD 4102, Australia; Louisiana State University Health Sciences center, UNITED STATES

## Abstract

The divergent TGF-β superfamily member, macrophage inhibitory cytokine-1 (MIC-1/GDF15), is overexpressed by most cancers, including prostate cancer (PCa). Whilst its circulating levels are linked to cancer outcome, the role MIC-1/GDF15 plays in cancer development and progression is incompletely understood. To investigate its effect on PCa development and spread, we have used TRAMP prostate cancer prone mice bearing a germline deletion of MIC-1/GDF15 (TRAMP^MIC-/-^). On average TRAMP^MIC-/-^ mice died about 5 weeks earlier and had larger prostatic tumors compared with TRAMP mice that were wild type for MIC-1/GDF15 (TRAMP^MIC+/+^). Additionally, at the time of death or ethical end point, even when adjusted for lifespan, there were no significant differences in the number of mice with metastases between the TRAMP^MIC+/+^ and TRAMP^MIC-/-^ groups. However, consistent with our previous data, more than twice as many TRAMP mice overexpressing MIC-1/GDF15 (TRAMP^fmsmic-1^) had metastases than TRAMP^MIC+/+^ mice (p<0.0001). We conclude that germ line gene deletion of MIC-1/GDF15 leads to increased local tumor growth resulting in decreased survival consistent with an overall protective role for MIC-1/GDF15 in early primary tumor development. However, in advancing disease, as we have previously noted, MIC-1/GDF15 overexpression may promote local invasion and metastatic spread.

## Introduction

Prostate cancer (PCa) is one of the most frequently diagnosed cancers in men. It caused an estimated 29,480 deaths in the USA in 2014 and is the second leading cause of cancer deaths in men [1]. Despite its clinical importance, our understanding of its biology is incomplete and aside from surgery for early stage disease, its therapy is palliative. Like most, if not all tumors, PCa displays altered expression of many gene products, including cytokines and growth factors. One cytokine commonly overexpressed in many cancers, including PCa, is MIC-1/GDF15, a divergent member of the transforming growth factor-β superfamily[2]. Expression of this cytokine is also induced by most cancer therapies and its serum levels are clearly linked to cancer outcome [3–9].

MIC-1/GDF15 is detectible in the blood of all individuals [10]. Its expression by cancers is frequently reflected by rises in its blood levels, usually in proportion to the stage and extent of tumor [5,11–16]. For example, there is a continuing rise in MIC-1/GDF15 serum levels with progression to colonic polyps, high grade dysplastic polyps, localized colorectal cancer (CRC) and then disseminated CRC [13]. Further, patients with CRC with elevated serum MIC-1/GDF15 levels at presentation, have a worse overall prognosis and earlier disease relapse [13,17]. For PCa, MIC-1/GDF15 serum levels are an independent predictor of the presence of cancer [14] and in more advanced disease they predict overall survival and bone metastasis [12,16]. High MIC-1/GDF15 serum levels also predict diagnosis and/or outcome for a wide range of malignancies including melanoma [18,19], cancers of the pancreas [15,20,21], thyroid [22,23], ovary [24] and endometrium [25].

In patients with advanced cancers, serum MIC-1/GDF15 levels commonly rise from a normal mean of about 450pg/ml [13] to 10,000–100,000 pg/ml or more [5] and may cause cancer anorexia/cachexia [26,27]. This common cancer complication is mediated by actions of MIC-1/GDF15 on feeding centres in the brain and can be reversed by neutralising antibodies [26,27]. MIC-1/GDF15 serum levels in cancer are influenced not only by its over-expression, but also depend on how it is processed by the tumor. Intracellular processing leads to removal of the MIC-1/GDF15 propeptide and diffusion into the blood stream after secretion. However, as the propeptide interacts with tumor stroma, unprocessed secreted protein remains bound to the extracellular matrix proximate to the producing tumor [21,28]. In PCa, increased stromal MIC-1/GDF15 is associated with better patient outcomes, especially in those with low-grade localized prostate tumors (Gleason sum score of 6 or less) [28], suggesting that its increased local availability is beneficial. By contrast, high circulating concentrations of MIC-1/GDF15 are associated with a poor outcome [28]. However, whether MIC-1/GDF15 overexpression in cancer has a beneficial, harmful or mixed effect on disease outcome is difficult to determine from epidemiological studies alone.

The *in vivo* cancer related activity of MIC-1/GDF15, has been examined in a number of tumor xenograft studies with mixed results. For example, enforced MIC-1/GDF15 overexpression in HCT-116 colon cancer cells [29] or in the DU145 [30] PCa cell line, xenografted into immunodeficient mice, reduced tumor size. A tumorigenic glioblastoma cell line, that remained unaffected by MIC-1/GDF15 *in vitro*, on transfection with MIC-1/GDF15, failed to develop tumors in nude mice [31]. The authors suggested that MIC-1/GDF15 may have acted on the local tumor microenvironment to inhibit tumor growth. By contrast, knock down of MIC-1/GDF15 in a human melanoma [18] and a mouse glioblastoma [32] cell line significantly decreased the growth of engrafted tumors. Further, the xenografts of PC3 PCa cell line engineered to overexpress MIC-1/GDF15 grew faster [33] and when orthotopically implanted, led to more metastases [34].

Unlike the xenograft models in immunodeficient mice, carcinogen induced and spontaneously developing cancer models are performed in immune competent mice, which more closely mimic the pathogenesis of cancers. In chemically induced cancer models, transgenic overexpression of MIC-1/GDF15 leads to resistance to urethane induced lung cancer [35] and azoxymethane induced colon cancer [36]. However, whilst transgenic overexpression led to protection in these two instances, gene deletion did not modify the development of diethylnitrosamine induced hepatocellular carcinoma [37].

Spontaneously developing cancers in transgenic mice often most closely conform to human cancers and all studies based on their use suggest that MIC-1/GDF15 is largely protective in early disease. Development of large bowel polyps and cancer in Apc^min^ mice is reduced by transgenic overexpression of MIC-1/GDF15 [36]. Further, germline deletion of MIC-1/GDF15 in Apc^min^ mice abolished the protection afforded from the COX inhibitor sulindac [38]. As this class of drug is known to induce expression of MIC-1/GDF15 in both mice and men [38], this data suggests that tumor suppression may be dependent on the expression of MIC-1/GDF15 [38]. Further supporting this view is a study utilising samples from the Polyp Prevention Trial [39]. This demonstrated that non-steroidal anti inflammatory drug (NSAID) users had a higher serum MIC-1/GDF15 level than non-users and only NSAID users with an elevated serum MIC-1/GDF15 level were protected from colonic adenoma recurrence [39,40].

More recently we have assessed the effect of MIC-1/GDF15 overexpression on the course of cancer in *T*ransgenic *A*denocarcinoma of *M*ouse *P*rostate (TRAMP) prostate cancer prone transgenic mice. TRAMP mice express SV40 early genes (T and t; Tag) under the control of rat probasin (rPB) promoter [41], which targets its expression to prostatic epithelium. Heterozygous TRAMP male mice develop progressive prostate cancer exhibiting the same spectrum of disease as found in men. Over the course of 6–12 months these mice progressively develop localized then invasive cancer that exhibits metastatic spread to distant sites, primarily the pelvic lymph nodes, liver, kidney and lungs [42]. Our data indicates that TRAMP mice, overexpressing MIC-1/GDF15, have substantially increased survival due to decreased growth and histological grade of the primary tumor [43], further supporting a beneficial role for MIC-1/GDF15 in early cancer. However, as the tumor advanced, these mice also developed more metastases [43], suggesting that MIC-1/GDF15 overexpression may have deleterious actions late in the course of cancer. There are no other data from transgenic cancer models where the effect of MIC-1/GDF15 on advanced cancers has been investigated.

It is important to understand the effect that MIC-1/GDF15 has on the biology of cancers as it is highly overexpressed by many cancers and its expression is induced by cancer therapies. Thus any effect it has on the biology of cancer is likely to be of clinical significance. To further advance our understanding of this cytokine in cancer, we have determined how MIC-1/GDF15 deficiency influenced the evolution of PCa. We have utilised TRAMP prostate cancer prone mice that also bear a germline deletion of the MIC-1/GDF15 gene (TRAMP^MIC-/-^) or wild type MIC-1/GDF15 (TRAMP^MIC+/+^), to compare survival rate, pattern of PCa growth and metastatic spread. TRAMP^MIC-/-^ mice had significantly larger prostate tumors and shorter survival than TRAMP^MIC+/+^ mice, but there was no significant difference in the incidence and rate of metastasis in the two mouse lines suggesting that different mechanisms mediate the effects of MIC-1/GDF-15 on local and metastatic PCa development. These data are consistent with earlier studies, identifying a largely protective role for MIC-1/GDF15 in the local growth of early cancers.

## Materials and Methods

### Ethics Statement

All research and animal care procedures were approved by the Garvan Institute/St Vincent’s Hospital Animal Experimentation Ethics Committee (Ethics No: 07/05, 10/05 and 13/08) and were in agreement with the Australian Code of Practice for the Care and Use of Animals for Scientific Purpose.

### Transgenic mice

Heterozygous male TRAMP mice (TRAMP^+/-^) [41] were generated by mating TRAMP^+/-^ females (C57BL/6 background) with non-transgenic C57BL/6 males. Mice with a germline deletion of the *MIC-1/GDF15* gene (MIC-1^-/-^) [44], also on a C57BL/6 background were bred with TRAMP mice to generate MIC-1^-/-^ mice also bearing the TRAMP transgene (TRAMP^MIC-/-^). The PB-SV40 T transgene was identified using DNA extracted from tail samples and PCR primers directed at the PB-SV40 T-antigen sequence: Pb-forward: 5’-CCGGTCGACCGGAAGCTTCCACAAGTGCATTTA-3’ and SV40Tag-reverse: 5’-CTCCTTTCAAGACCTAGAAGGTCCA-3’. MIC-1/GDF15 gene deletion was identified using primers MIC1Exon2for: 5’-GGCGGCGCACAGCTGGAACTGC-3’ with MIC1Exon2Rev: 5’-CAGCCCCGGGCCACCAGGTCAT-3’ (Wild type pair) and MIC-1/GDF15KOfor: 5’-GAGAGGACTCGAACTCAGAACCA-3’ with MIC-1/GDF15KORev: 5’-GAAGTTATATTAAGGGTTCCGCAAGC-3’ (Knock-out pair). Syngeneic mice overexpressing MIC-1/GDF15 under control of the myeloid cell specific c-fms promoter (MIC-1^fms^) were used to breed TRAMP mice that also overexpress MIC-1/GDF15 (TRAMP^fmsmic-1^). The double transgenic TRAMP^fmsmic-1^ mice were generated by crossing TRAMP^+/-^ females with homozygous MIC-1^fms^ males. The MIC-1/GDF15 transgene in TRAMP^fmsmic-1^ mice was identified by PCR using primers, Flag-forward: 5’-GACTACAAGGACGACGATGACAAG-3’ and MS8-reverse: 5’-CGAAGCCTACCGCGTGCACCGAG-3’. The reaction conditions used were: denaturation at 95°C for 10 s, annealing at 60°C for 20 s, and extension at 72°C for 30 s.

### Survival study

Based on a statistical power analysis for sample size, (Alpha error = 0.05, statistical power = 0.95), 35 TRAMP^MIC+/+^ and 35 TRAMP^MIC-/-^ mice were allocated at 4–6 weeks of age, for a survival study. From that time, mice were weighed once a week and monitored twice a week for tumor size and extent by palpating the abdomen. Mice either died or were culled when they reached ethical end points of tumor size larger than 11mm X 11mm X 11mm, more than 20% weight loss or meeting any other ethical end point criteria for euthanasia. The overall survival of individual mice was calculated from birth to ethical end point or death from the tumor. Survival distribution was estimated using the method of Kaplan-Meier. At necropsy the genitourinary complex (GU) consisting of prostate (including dorsal, lateral, ventral, and anterior lobes), urethra, ampullary gland, seminal vesicle (SV) and urinary bladder was taken out and weighed. Prostate was excised from GU and weighed separately. Weight of the GU and prostate of each mouse was normalized by its body weight (organ wt/body wt).

### Primary tumor size

In a separate cohort to that above, prostate tumor growth was compared in TRAMP^MIC+/+^ and TRAMP^MIC-/-^ mice. At the start of the study 88 TRAMP and 88 TRAMP^MIC-/-^ mice, 22 of each for each stage, were pre-allocated to be sacrificed at different time points from early to advanced tumor stages (8, 17, 25 and 33 weeks of age). For each of the 88 mice necropsied, the GU was excised and prostate was separated from GU. Total GU and prostate weight were recorded and normalized for the donor mouse total body weight (organ wt/body wt).

### Identification of tumor metastases

To estimate the occurrence of metastasis at the time of death or culling in TRAMP^MIC+/+^ and TRAMP^MIC-/-^ mice, examined a different cohort of TRAMP^MIC+/+^ (n = 63) and TRAMP^MIC-/-^ (n = 63). For comparison, we also examined a similar number of MIC-1/GDF15 overexpressing TRAMP^fmsmic-1^ mice (n = 63), whose PCa was known to be associated with increased metastases [43]. Mice were looked after and euthanized using the same criteria as mentioned above in the survival study. At the necropsy pelvic lymph nodes, kidney, and liver tumors (if present) were harvested and fixed in 10% neutral buffered formalin. Lungs were excised, weighed and fixed in Bouin’s fixative (Sigma-Aldrich) to visualize and count lung tumor colonies. Metastatic lesions on all the organs were counted under a dissecting microscope. Some of the lesions were confirmed by H&E staining and further by immunostaining of frozen tissue sections with anti Tag antibody (Santa Cruz) to confirm the prostatic origin of the tumor. The number of mice having distant organ metastasis was compared in all the three mouse lines.

### Statistical analysis

All graphs and statistical evaluation of all the experiments were performed with GraphPad Prism software version 6 for Mac OS X, (GraphPad Software, San Diego, CA, USA). Kaplan–Meier analysis and log-rank statistic were used to compare survival curves. The data for GU and prostate tumor sizes between groups were compared using 2-way ANOVA or t-test. The Chi-square test was used for categorical analysis. Statistical power analysis for sample size was done with the online power and sample size analysis tool (http://www.stat.ubc.ca/) using an Alpha Error of 0.05 and statistical power level of 0.95. Multivariate logistic regression analysis was used to examine the relationship of metastasis with survival time. A p value less than 0.05 considered statistically significant.

## Results

### MIC-1/GDF15 gene deleted TRAMP mice die earlier of PCa

**Fig 1:**
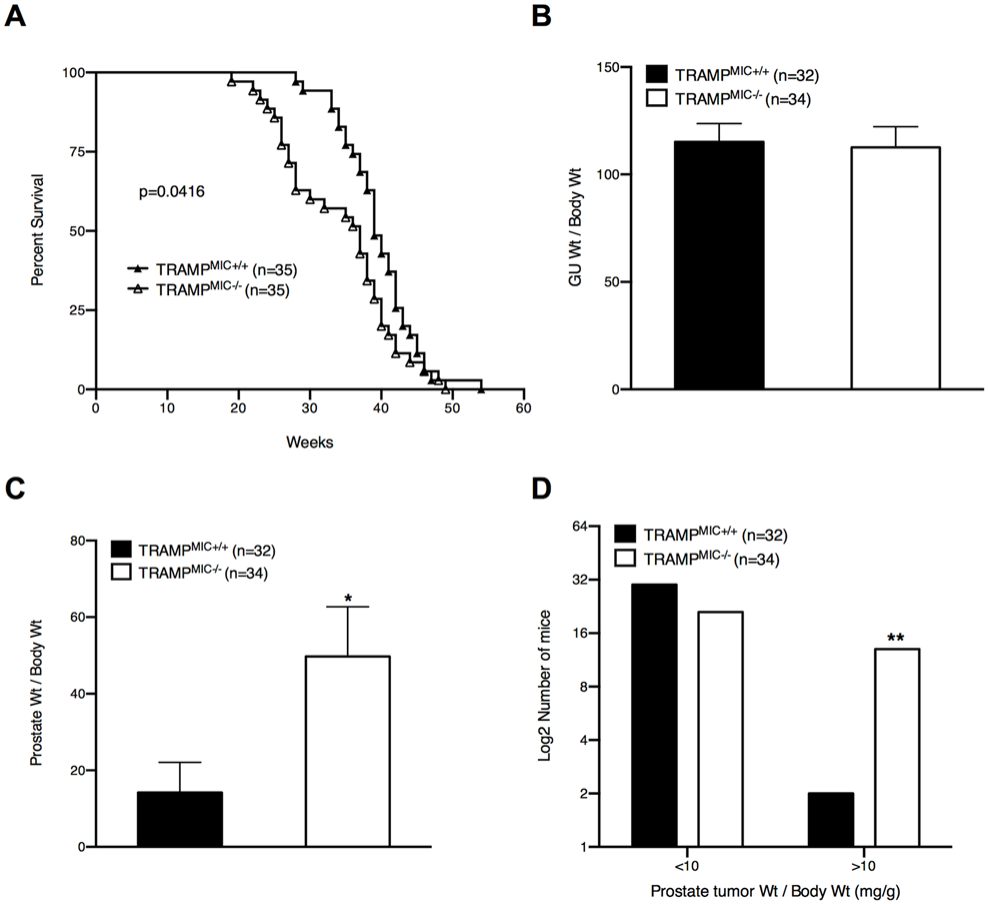
TRAMP^MIC-/-^ mice have shorter survival and larger prostate tumors than TRAMP^MIC+/+^ mice. (A) Survival data for TRAMP^MIC+/+^ and TRAMP^MIC-/-^ mice (n = 35). Overall survival of individual mice from birth to death was plotted using the Kaplan-Meier method. The log-rank statistic for median survival time is shown. (B) The genitourinary complex (GU) and prostate tumor weights (C), in TRAMP^MIC+/+^ and TRAMP^MIC-/-^ mice, at the necropsy, are corrected for body weight and presented as mean ± SEM. Differences are analyzed using an unpaired 2-tailed t test. (D) The number of TRAMP^MIC+/+^ and TRAMP^MIC-/-^ mice having large prostate tumor (corrected tumor wt>10mg/g), was compared using a Chi-square test. p values are shown as *, p*<* 0.05; **, p*<* 0.01.

In order to assess the effects of MIC-1/GDF15 gene deletion on the overall survival of TRAMP mice, we monitored a cohort (n = 35) of TRAMP^MIC+/+^ and TRAMP^MIC-/-^ mice (Survival Group) till death or ethical end point. Kaplan-Meier survival analysis showed that TRAMP^MIC-/-^ mice had significantly shorter survival than TRAMP^MIC+/+^ mice (Fig. 1A, p *=* 0.0416, log-rank test). The mean survival of 39.5 weeks in TRAMP^MIC+/+^ mice was reduced by about 5 weeks (34.4 weeks) in the TRAMP^MIC-/-^ group. Further, while only 20% of TRAMP^MIC-/-^ mice survived at week 40, 42.85% of TRAMP^MIC+/+^ mice were still alive (Fig. 1A). These data indicate that germline gene deletion of MIC-1/GDF15 reduced PCa related survival in TRAMP mice.

### TRAMP^MIC-/-^ mice have larger prostate tumors at necropsy

At the necropsy of the above-mentioned survival group of TRAMP^MIC+/+^ and TRAMP^MIC-/-^ mice, GU and prostate were isolated and their weights, corrected for body weight, were recorded. Despite dying earlier than TRAMP^MIC+/+^ mice, TRAMP^MIC-/-^ mice on average had significantly heavier prostate tumors at the time of death (Fig. 1C, p = 0.0240). Further, the TRAMP^MIC-/-^ group had far more mice with very large prostate tumors (normalized prostate weight >10mg/g) than the TRAMP^MIC+/+^ group (Fig. 1D, p = 0.0027, Chi-square test). There was no significant difference in total GU wt (Fig. 1B) between two mouse lines because TRAMP^MIC+/+^ had significantly larger SV tumors than TRAMP^MIC-/-^ mice (data not shown). This data suggests that deletion of *MIC-1/GDF15* gene was associated with increased local prostate tumor growth in TRAMP mice, perhaps with reduced seminal vesicle invasion.

### MIC-1/GDF15 deletion enhances PCa growth in TRAMP mice

**Fig 2:**
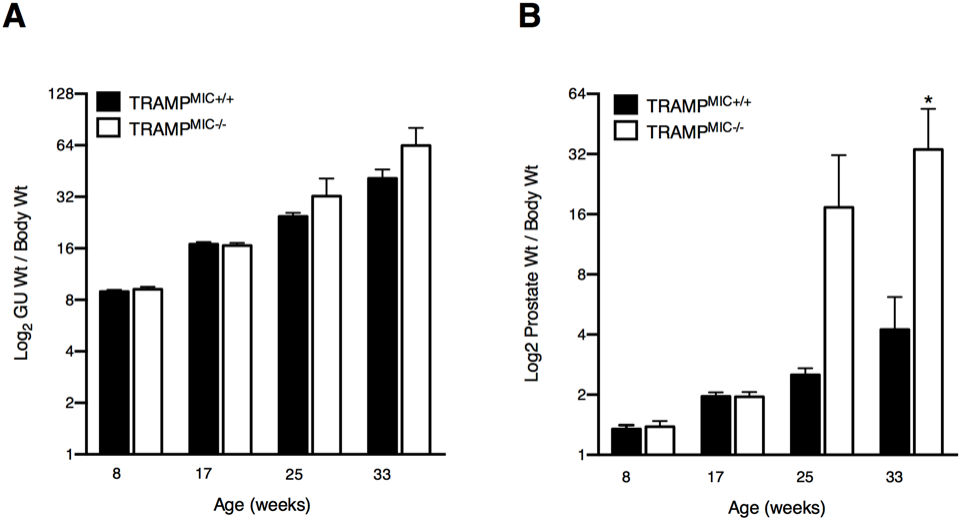
TRAMP^MIC-/-^ mice have comparatively larger prostate tumor than TRAMP^MIC+/+^ mice. The corrected tumor weights of (A) GU and (B) prostates were compared in TRAMP^MIC+/+^ and TRAMP^MIC-/-^ mice (n = 22/group/time point) sacrificed at 8, 17, 25 and 33 weeks of age. Results were analyzed using a 2 way ANOVA and are presented as mean ± SEM. p values are shown as *, p*<* 0.05.

To further assess the impact of MIC-1/GDF15 on prostate cancer growth, at 4–6 weeks of age, we pre-assigned another cohort of 88 TRAMP^MIC+/+^ and 88 TRAMP^MIC-/-^ mice to be culled progressively at four predefined time point up to 33 weeks of age. Consistent with the data from the survival study group mice, discussed above, there was no significant difference in the normalized GU weights between two mouse lines at any time point observed (Fig. 2A). When we looked at the prostate size, there were no detectible differences in the average corrected prostate weights between TRAMP^MIC+/+^ and TRAMP^MIC-/-^ mouse lines at week 8 and 17. At week 25 and 33, TRAMP^MIC-/-^ mice had a 6.9 and 8 fold increase in corrected average prostate tumor weight respectively, as compared to TRAMP^MIC+/+^ mice (Fig. 2B) and this difference was statistically significant at week 33 (p = 0.0098, 2 way ANOVA).

### *MIC-1/GDF15* gene deletion has no effect on metastases

**Fig 3:**
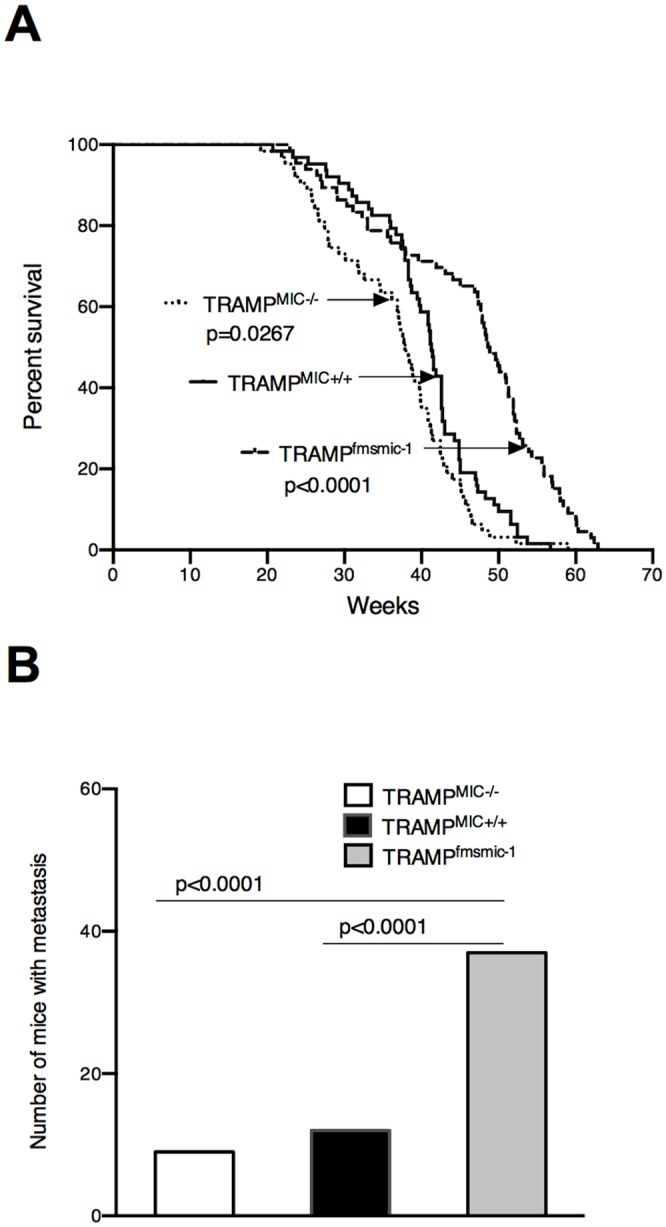
Effect of *MIC-1/GDF15* gene modification on metastases. (A) Survival data for TRAMP^MIC+/+^ (n = 63), TRAMP^MIC-/-^ (n = 63) and TRAMP^fmsmic-1^ (n = 63) mice is presented as a Kaplan-Meier plot and the log-rank statistic for median survival time is shown. (B) Comparison between number of TRAMP^MIC+/+^ (n = 63), TRAMP^MIC-/-^ (n = 63) and TRAMP^fmsmic-1^ (n = 63) mice having distant organ metastases at the time of death has been analyzed using the Chi-squared test.

Since metastasis is the major cause of death in patients with human PCa, we evaluated the effect of *MIC-1/GDF15* gene deletion on the incidence and extent of metastasis in TRAMP mice. We examined a separate cohort of 63 TRAMP^MIC+/+^, 63 TRAMP^MIC-/-^ and 63 MIC-1/GDF15 overexpressing TRAMP (TRAMP^fmsmic-1^) mice, which were followed until death or ethical end point. The latter group was included as a positive control, as our previous study had indicated TRAMP^fmsmic-1^ mice have more metastases but survive longer [43]. Kaplan-Meier survival analysis reconfirmed that TRAMP^MIC-/-^ mice die significantly earlier than TRAMP^MIC+/+^ mice (Fig. 3A, p *=* 0.0267, log-rank test), consistent with data in the survival group. Further, TRAMP^fmsmic-1^ mice died at significantly slower rate than TRAMP^MIC+/+^ mice (Fig. 3A, p<0.0001, log-rank test) confirming data from our previous publication [43]. In this cohort 19% of the TRAMP^MIC+/+^ mice developed macroscopically detectible distant organ metastasis in the surveyed organs, which was not significantly different to that of 14.2% in TRAMP^MIC-/-^ mice (Fig. 3B). The incidence of metastases in these two mouse lines was significantly less that in TRAMP^fmsmic-1^ mice, 59% of which developed metastases (Fig. 3B). These data show that although TRAMP^MIC-/-^ mice die significantly earlier than TRAMP^MIC+/+^ mice (Fig. 3A) there were no significant differences in the incidence of distant organ metastasis between the two mouse lines (Fig. 3B). In contrast, as previously reported, a significantly higher proportion of TRAMP^fmsmic-1^ mice showed distant organ metastasis compared with TRAMP^MIC+/+^ or TRAMP^MIC-/-^ mice (Fig. 3B, p<0.0001, Chi-square test). Multivariate logistic regression analysis confirmed that the increased proportion of TRAMP^fmsmic-1^ mice with metastases was independent of their longer survival times (p>0.5) and only dependent on genotype (p<0.0001). Further, using a similar approach, the lack of difference in the proportion of TRAMP^MIC-/-^ compared with TRAMP^MIC+/+^ mice with metastases, could not be accounted for by their shorter survival (p>0.5).

## Discussion

This study clearly indicates that germline deletion of the MIC-1/GDF15 leads to increased local primary tumor growth resulting in earlier death of TRAMP PCa prone mice. These data are consistent with our previous publication indicating that transgenic overexpression of MIC-1/GDF15 decreases local PCa tumor growth and substantially increases survival of TRAMP mice. This result is consistent with results in another transgenic model of early cancer in Apc^min^ colonic polyp prone mice also overexpressing MIC-1/GDF15 [38]. This reinforces the argument that MIC-1/GDF15 plays a protective role in early local tumor development and growth. Several epidemiological studies suggest that this finding may be translated to at least some human cancers. For example, increased local concentrations of extracellular matrix associated MIC-1/GDF15 in prostate cancer biopsies were associated with reduced risk of disease progression, especially in the subgroup with early cancer and with Gleason grade of 6 or less. In this group MIC-1/GDF15 localised to the tumor matrix was the single best predictor of tumor recurrence [28]. In patients from the polyp prevention trial, a higher polyp free serum MIC-1/GDF15 level afforded protection from polyp recurrence and NSAID mediated protection was lost if MIC-1/GDF15 serum levels were not elevated with NSAID treatment [39].

The apparently discordant effect of MIC-1/GDF15 on local tumor growth and the metastatic process, that we described previously, is again demonstrated here [43]. Transgenic overexpression leads to smaller local tumors and longer survival, as TRAMP^cfmsmic-1^ mice no longer die early of local disease. Independently of increased survival time, these same TRAMP^cfmsmic-1^ mice have more metastases. However, we could demonstrate no difference in the proportion of mice with metastases between TRAMP^MIC+/+^ and TRAMP^MIC-/-^ mice. As few mice of either line developed metastases by the experimental endpoint we may not have studied enough mice to be certain that no differences exist. Additionally, as prostate tumors in TRAMP^MIC+/+^ mice express little MIC-1/GDF15 [43], it may be that this model is more sensitive to the effects of increased expression compared to gene deletion.

Whilst there was no significant difference in metastases between TRAMP^MIC+/+^ and TRAMP^MIC-/-^ mice, there may be differences in local invasion. TRAMP^MIC-/-^ mice with larger prostate tumors had smaller SVs (data not shown), which suggests that there may be less SV invasion.

How MIC-1/GDF15 might exert effects on tumor growth and spread is uncertain. Elucidating its mechanisms of action is greatly hampered because the identity of its receptor is unknown. It is presumed that the receptor is a member of the highly conserved hetrotetrameric TGF-b receptor (TBR) superfamily, but there is limited direct evidence for this. There is some indirect evidence, based on antibody blockade, that it may utilise TBRII [26,45] the class 2 receptor used by TGF-b itself, but there is no direct biochemical or genetic evidence supporting this data. The signalling cascade of the receptor complex or class I receptor utilised by MIC-1/GDF15 is to date, completely unknown.

There is now a plethora of published data on the role of MIC-1/GDF15 on tumor growth and spread with a confusing range of results. Data from experiments using human cell lines, in which MIC-1/GDF15 expression has either been induced or knocked down, then xenografted into immunodeficient mice, have provided sometimes, contradictory results [18,29–34]. Studies using transgenic models of spontaneously developing cancer that also bear genetically modified MIC-1/GDF15 expression and an intact immune system all point to a protective role for MIC-1/GDF15 on local tumor development. A similar effect can also be observed in two models of carcinogen induced cancer in transgenic mice overexpressing MIC-1/GDF15, which also have an intact immune system [35,36]. The most parsimonious explanation that may explain these contradictions is that MIC-1/GDF15 regulates anti-cancer immunity, which in turn regulates cancer growth.

Overall, our results support an important protective role for MIC-1/GDF15 in the development and early growth of PCa and probably cancer in general. Unravelling the biological effect of MIC-1/GDF15 on tumor evolution and biology is of practical importance for several reasons. A high proportion of cancers express it, to the extent that serum level can rise up to 10–100 fold and cause cancer anorexia/cachexia. Further, its expression is increased by all cancer treatment modalities including surgery, radiotherapy and chemotherapy. Thus any effect that MIC-1/GDF15 has on local tumor biology, particularly tumor spread is likely to impact most cancer patients, raising the prospect that modulation of MIC-1/GDF15 actions during therapy might reduce the risk of metastatic disease and other complications of cancer.
